# GravSorter: a forward-genetics tool for studying gravity response in *Caenorhabditis elegans*

**DOI:** 10.1039/d5an01231g

**Published:** 2026-04-20

**Authors:** Hui Ma, David M. Raizen, Haim H. Bau

**Affiliations:** a Department of Mechanical Engineering and Applied Mechanics, University of Pennsylvania Philadelphia PA USA bau@seas.upenn.edu; b Department of Neurology, Perelman School of Medicine, University of Pennsylvania PA USA

## Abstract

*Caenorhabditis elegans* exhibits multiple forms of taxis that couple sensation modalities to directed locomotion. Dissecting the genetic architecture underlying these behaviors requires scalable, high-throughput screening tools that convert locomotory biases into robust, selectable phenotypes. Here, we introduce GravSorter, a forward-genetics platform designed to identify genes required for gravitaxis. GravSorter comprises a fluidic system with vertical columns filled with a buffer slightly denser than the worms. During positive gravitaxis, taxis-competent animals actively orient and swim downward, overcome buoyancy, and are collected at the bottom of the columns. Taxis-deficient worms lack directional swimming bias, rise to the top, and are collected there for re-sorting to increase selection stringency. Previously, we showed that wild-type *C. elegans* exhibits positive gravitaxis, whereas the dopamine-deficient mutant *cat-2* does not. GravSorter performance was evaluated by separating gravitaxis-deficient *cat-2* mutants from wild-type controls and by distinguishing *cat-2* mutants from pharmacologically rescued *cat-2* animals. GravSorter provides an efficient and generalizable platform for identifying genes and neural circuits that govern directed locomotion in response to environmental stimuli and for assessing drug efficacy. The underlying principle, opposing active taxis-driven thrust with a passive taxis-independent force, provides a generalizable framework for high-throughput forward genetic screens to investigate diverse taxis modalities and their underlying neural circuits.

## Abstract 2

GravSorter: Diverse genotypes are placed in a vertical liquid column slightly denser than the worms. Gravitaxis-competent worms swim downward, while deficient worms float and are collected for downstream sorting and sequencing.

## Graphical abstract

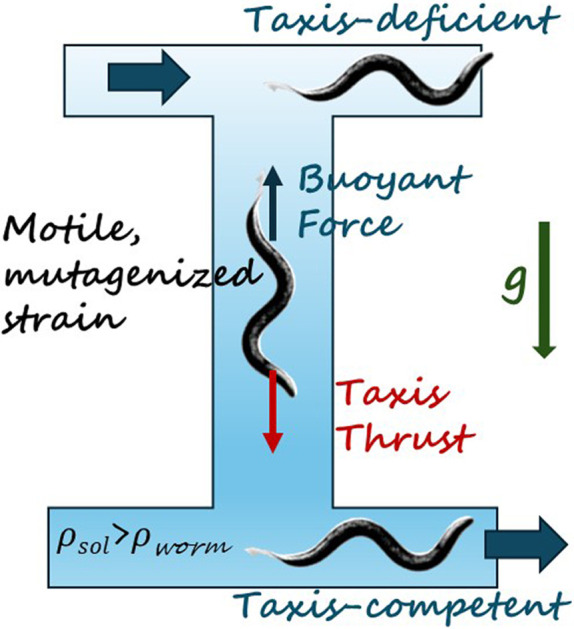

## 1. Introduction

**Fig. 1:**
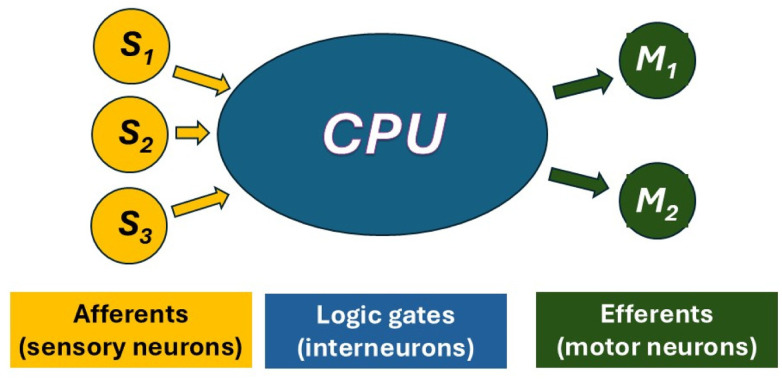
Schematic depiction of the neural circuit that detects, processes, and generates behavioral responses to environmental stimuli. Defects in any circuit component or interference from competing sensory inputs can result in taxis deficiency.

The experimental model organism *Caenorhabditis elegans* (*C. elegans*), with its simple, well-characterized nervous system and extensive genetic toolkit, is a powerful model for investigating the mechanisms underlying animal behavior. A major advantage of *C. elegans* is its suitability for forward genetic screens: animals carrying random mutations are screened for a phenotype of interest, and the mutated genes responsible for these phenotypes are subsequently identified, providing mechanistic molecular insight. Importantly, a substantial fraction of *C. elegans* genes have human homologs,^1^ and many conserved molecular pathways govern neural function and behavior across species. Thus, discoveries in *C. elegans* frequently provide insight into gene function and biological processes relevant to human health and disease. To dissect the circuits responsible for taxis (Fig. 1), it is necessary to efficiently isolate taxis-deficient mutants and then identify the causative mutations. In addition to enabling analysis of genotype–phenotype relationships, this forward genetic approach provides insight into the underlying neural circuitry by identifying the anatomical and cellular sites at which genes act to control taxis behavior.

To accomplish this, we propose a sorting strategy in which motile worms within a column are exposed to a taxis stimulus applied at one end, eliciting deliberate directional swimming toward that end. Simultaneously, the worms experience an opposing passive force, weaker than their propulsive thrust, directed toward the opposite end. Taxis-deficient worms, which lack a sustained directional bias, are carried by the passive force and collected at one end of the column, whereas taxis-competent worms actively overcome this force and accumulate at the opposite end.

**Fig. 2:**
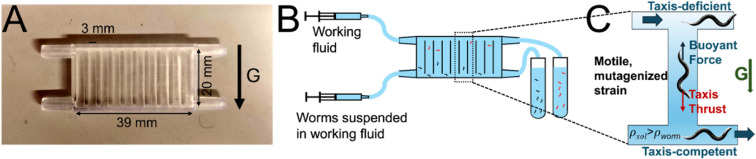
(A) The sorter is 3D-printed. It has 10 columns, each serving as a single sorting unit. (B) The liquid flow in the sorter is controlled by a double-barreled (dual) syringe pump, which pumps fluid from left to right at a rate that cannot be overcome by worm rheotaxis.^2–5^ The worm suspension is introduced through the bottom inlet. The two syringes induce the same flow rate. The aqueous fluid bathing the animals is slightly denser than the worms’ density. The worm population collected at the top right outlet is enriched for gravitaxis-deficient animals, and the worm population collected at the bottom right outlet is enriched for gravitaxis-competent worms (Video S1). (C) Operating principle of the sorter. Taxis-competent worms (*e.g.*, during gravitaxis) produce a directed propulsive thrust that overcomes an imposed opposing force, such as buoyancy, enabling their separation from taxis-deficient worms within a mixed population.

To identify circuit components required for positive gravitaxis—the focus of this study—a vertical column is filled with a buffer slightly denser than the worms (Fig. 2). Gravitaxis-competent worms swim downward against buoyancy and are collected at the bottom, while gravitaxis-deficient worms, lacking directional preference, passively rise and accumulate at the top. This sorting principle can be generalized to other forms of taxis, in which the passive counterforce is generated by controlled fluid flow rather than buoyancy.

We use 3D printing to fabricate a sorter capable of efficiently processing thousands of worms. Microfluidic platforms have been widely applied in *C. elegans* research^2,6–8^ for high-throughput manipulation, screening, and behavioral analysis, highlighting their value for large-scale genetic studies. Our sorter (Fig. 2 and Video S1) consists of vertical columns connected at their bottoms to a feed line and at their tops to a collection line. The system is filled with an aqueous working fluid slightly denser than the worms. A mixed population of worms is introduced through the bottom feed line. Gravitaxis-deficient worms, which lack sustained directional swimming, float to the top of the columns and exit through the collection line for further analysis or re-sorting. In contrast, gravitaxis-competent worms actively swim downward against buoyant forces, remain near the bottom, and are subsequently drained from the sorter.

## 2. Methods

### 2.1 Sorter design

Our gravitaxis sorter consists of ten identical vertical columns, each measuring 18 mm in height with a 3 mm × 3 mm cross-section (Fig. 2). These columns are connected at their tops to a horizontal collection line and their bottoms to a feed line, each with a cross-section of 1 mm × 3 mm. The entire sorter has a width of 41 mm, a height of 22 mm, and a depth of 5 mm. The device is 3D-printed (FormLabs 4™) with clear V4 resin. The feed and collection lines connect, *via* Luer-slip fittings, to a dual-barrel syringe pump (PHD 2000, Harvard Apparatus). The pump is adjusted to deliver flow in each line at an average cross-sectional velocity of 0.5 mm s^−1^ – sufficient to prevent the worms from swimming upstream.^2–5^ Sorted worms exit through the outlet port into a 15 mL collection tube and through the bottom port into a waste tube. Following sorting, worms are transferred from the tubes into agar plates and imaged under a stereomicroscope. Worm counts were performed using a custom MATLAB script.

We selected the vertical column dimensions to minimize secondary flows induced by the feed and collection lines (section 3.2). The working fluid consisted of a blend of M9 buffer and a biocompatible aqueous suspension of 12 nm diameter colloidal silica particles (LUDOX HS-40, Sigma; density 1.3 g mL^−1^ at 25 °C (ref. 9) mixed at a 1 : 0.315 volume ratio, yielding a solution density of 1.09 g mL^−1^. This density is slightly greater than that of the worms (∼1.08 g mL^−1^), causing gravitaxis-deficient worms to undergo buoyant rise. In contrast, gravitaxis-competent worms overcome this buoyancy by swimming downward (section 3.1). The density of the working fluid was measured by weighing 1 mL of the solution. At this concentration, the suspension behaves as a homogeneous Newtonian fluid with a viscosity approximately 4.78 times that of water.^9^

To adjust the working fluid density and observe the behaviors of individual worms, we used an auxiliary apparatus consisting of a vertical 200 mm tall cuvette with a 10 mm × 10 mm square cross-section, illuminated from below with red light (Video S1). Two cameras (DFK37AUX226, Imaging Source), mounted orthogonally (90*°* apart) at the same height, monitored the worms’ orientations concurrently in the *x*–*z* and *y*–*z* planes, where *z* is the vertical direction. Videos were recorded at 16 frames per second.

The absence of significant flow in the vertical columns is critical to our sorter's successful operation. To verify that there was no crossflow between the bottom feed conduit and top collection conduit, we filled one syringe with red dye and the other with blue dye (Pitsco Education) to monitor the flow in the sorter. The exit ports discharged either blue or red fluid exclusively. No green (mixed) fluid was observed, indicating minimal crosstalk. Additionally, we measured the liquid volumes collected in the two collection tubes during a sorting run and found them to be within 3%, further indicating the absence of significant fluid flow in the vertical columns.

### 2.2 Worm husbandry and preparation

Worm maintenance followed standard practices (Stiernagle, WormBook^10^). We used hermaphrodites in all experiments. Worms were cultivated at 20 °C on NGM agar Petri dishes (5.5 cm diameter) seeded with a lawn of *Escherichia coli* bacteria (strain DA837^11,12^) until they reached adulthood and produced eggs. Three milliliters of M9 buffer (3 g KH_2_SO_4_, 6 g Na_2_HPO_4_, 5 g NaCl, 1 mL 1 M MgSO_4_, and pure water to 1 L) were added to the plates to wash off the egg-harboring adults. The worm suspension was transferred to a 15 mL tube containing M9 buffer and centrifuged at 3000 rpm for 1 minute. The supernatant was decanted. Ten milliliters of 10% bleach solution (Germicidal Bleach, Clorox) was added to the tube to dissolve the collagenous cuticles of adult worms, while the eggs remained protected by their chitinous cuticles. After 10–15 minutes, the worm bodies were fully dissolved. The tube was refilled with M9 buffer, centrifuged, and the supernatant decanted. We repeated the washing and centrifugation steps three times to remove residual bleach. The eggs were then transferred with a serological pipette onto the surface of fresh NGM agar plates seeded with bacteria. We performed experiments 3 days later, on day-1 adult worms. We washed the adult worms off the plates using M9 buffer and briefly (∼30 seconds) centrifuged them to separate the worms from the bacteria. For the sorting experiment, we transferred the worms into our working fluid, which had a density of 1.09 g mL^−1^. Unless noted otherwise, the time elapsed from floating the worms off their cultivation plate to collecting the sorted animals was 10–30 minutes.

We used the *C. elegans* strains: N2 (wild-type, WT);^13^ NQ1155,^14^ which is an N2 strain carrying an extrachromosomal transgene encoding green fluorescent protein (GFP)^14^ in pharyngeal muscle; PD4790, which is an N2 strain carrying an integrated transgene mIs12 [myo-2p::GFP + pes-10p:GFP + F22B7.9::GFP] II, expressing a GFP reporter in multiple tissues; and MT15620, which harbors a *cat-2* (*n4547*)II gene deletion, which we confirmed by PCR (SI, section S1). *cat-2* encodes tyrosine hydroxylase, which catalyzes the rate-limiting step in dopamine biosynthesis.^15^ N2, PD4790, and MT15620 were all obtained from the CGC. NQ1155 was made by the Raizen lab.

### 2.3 Pharmacological rescue

We performed a pharmacological rescue using l-DOPA (3,4-dihydroxy-l-phenylalanine; Sigma-Aldrich, catalog no. 59-92-7). One group of *cat-2* mutant worms was cultured on agar plates supplemented with 10 mM l-DOPA for 72 hours prior to the experiment. A second rescue group was treated with 10 mM l-DOPA plus 1 mM l-(+)-ascorbic acid (Fisher Scientific, catalog no. AAA1561318) to limit l-DOPA oxidation. A third group received only 1 mM ascorbic acid alone as a vehicle control. A fourth group was exposed to 10 mM l-DOPA for 1 hour after reaching adulthood to assess the acute effects of dopamine replacement.

After incubation on drug-doped plates, we removed the worms and washed them with M9 buffer containing 10 mM l-DOPA. The working fluid, with the same density as described earlier, was prepared by dissolving 10 mM l-DOPA. For the control group, the working fluid did not contain any l-DOPA.

### 2.4 Gait frequency measurement

To assess whether sorted worms were mobility impaired (unable to produce propulsive thrust), we compared the ventrodorsally bending frequency and amplitude of wild-type worms collected from our sorter's top and bottom outlets. We transferred worms suspended in the working liquid onto agar plates seeded with bacteria to provide a food source and added 3 mL of M9 buffer to form a 3 mm-high liquid layer above the agar. We then recorded videos of the swimming worms and analyzed their undulatory motion using ImageJ. For each of the 30 worms collected from the top and bottom outlets, we measured the duration of five complete undulatory cycles and calculated the average cycle period. The inverse of this period is the gait frequency.

### 2.5 Computations of the flow field in the sorter and modeling the sorter's performance

To gain further insight into our sorter's operation and to assess potential adverse effects of secondary flows at the junctions between the vertical columns and horizontal conduits, we solved the steady-state Navier–Stokes equations for an incompressible, Newtonian fluid using the finite element Computational Fluid Dynamics (CFD) software COMSOL Multiphysics (version 6.2).

**Fig. 3:**
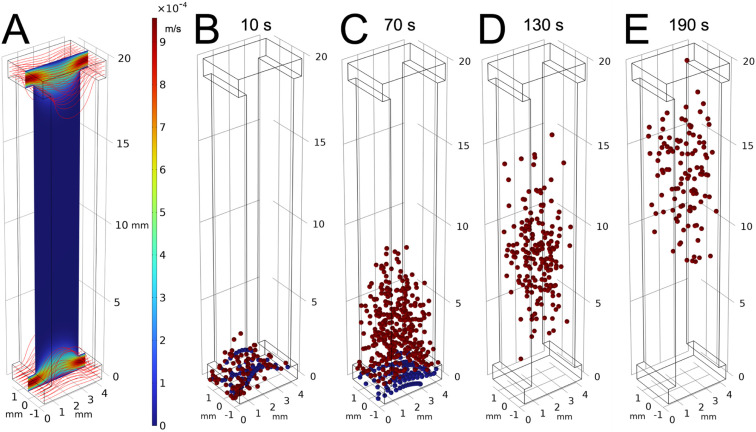
Computer simulation of the sorter function. The working fluid is introduced at the two inlets at the same flow rate. (A) There are slow vortices in the columns, which are too weak to trap the worms. The color bar indicates velocity magnitude in m s^−1^. The red curves are streamlines. (B–E). Gravitaxis-proficient (blue) and deficient (red) particles, having the same propulsive thrust, are released at the bottom. The red (gravitaxis-deficient) particles reorient their swimming direction randomly and float to the top by buoyancy. The blue particles, being gravitaxis-competent, sense the direction of gravity and actively swim downward. Thus, the gravitaxis-deficient particles are separated from the gravitaxis-competent particles (Video S2).

The fluid was assigned properties matching our LUDOX blend (density *ρ*_f_ = 1.09 g cm^−3^, dynamic viscosity *µ* = 0.00478 Pa s). The computational domain consisted of a representative vertical column (Fig. 3A) and adjoining segments of the horizontal top collection and bottom waste conduits, extending from the mid-distance between the upstream column and the mid-distance between the downstream column, replicating the geometry of our 3D-printed device (Fig. 2C). No-slip and impermeability conditions were applied to all solid boundaries. At the inlets and outlets of the horizontal conduits, we imposed a fully developed velocity profile with a mean velocity of 0.5 mm s^−1^. To ensure uniqueness, the pressure was fixed at a single interior point within the computational domain.

We computed particle trajectories using the Particle Tracer Module in COMSOL, which integrates the equations of motion to update particle positions at each time step. A total of 600 “gravitaxis-competent” particles (blue) and 600 “gravitaxis-deficient” particles (red) were introduced into the computational domain through the bottom feed conduit.

## 3. Results

### 3.1 Worm propulsive thrust

Our sorting method requires the working fluid to be denser than the worms to generate an upward buoyant force, but not so dense as to counteract the propulsive thrust of gravitaxis-competent worms deliberately swimming downward. To estimate this thrust, we performed experiments using a vertical cuvette filled with M9 buffer (density ∼1.0 g mL^−1^) and tested wild-type N2 and *cat-2* (MT15620) worms.

Worms were introduced just below the liquid surface to avoid surface tension effects. Because the worms are slightly denser than the M9 buffer, they sedimented toward the bottom (SI Video S1). Gravitaxis-competent worms (N2) oriented headfirst in the direction of gravity and swam downward (Video S1).^6^ In contrast, *cat-2* mutants displayed random body orientations and swam in all directions.^6^

We approximate each worm as a rigid spherical particle moving at a velocity *U* relative to the surrounding viscous fluid. In other words, *U* denotes the velocity difference between the worm's center of mass and the local fluid. Because worm motion is at low velocity (low Reynolds number, Re < 0.1), inertial effects are negligible, and the forces on the particle are in quasi-static balance. The forces acting on the particle are: (i) propulsive thrust (*F*_p_) generated by the worm's swimming, (ii) gravitational force (*ρ*_w_ − *ρ*_f_)*V*_w_*g*, and (iii) viscous drag given by Stokes law 6π*µr*_s_*U*. Here, *µ* and *ρ*_f_ ∼ 1.0 g cm^−3^ are, respectively, the dynamic viscosity and density of the bathing liquid. *r*_s_ is the particle's Stokes radius, determined empirically so that the particle experiences the same drag as the worm. *V*_w_ ∼ 5.96 × 10^−12^ m^3^ (ref. 16) is the effective worm volume. *g* is the gravitational acceleration. The Reynolds number Re = *ρ*_f_*Ur*_s_/*μ*. Force balance requires:

1 (*ρ*_w_ − *ρ*_α_)*V*_w_*g* + 6π*µr*_s_*U* + *F*_p_ = 0

We assume N2 and *cat-2* worms have similar volumes, densities, and Stokes radii (section S2).

The *cat-2* mutants reorient their swimming direction randomly with a characteristic reorientation time (mean time over which direction persists) of *τ*_r_ = 4.65 ± 1.95 s (*N* = 32). Consequently, the gravitaxis-deficient worms behave like active Brownian particles experiencing Stokes drag with drag coefficient *C*_f_ = 6π*µr*_s_ and randomly oriented drift velocity *U* = *F*_thrust_/*C*_f_ (in the absence of buoyancy). The apparent diffusion coefficient due to this randomly oriented propulsion is approximately *D*_app_ = *U*^2^*τ*_r_/3.^17^ Because *cat-2* worms continually change direction, their self-propulsion does not contribute to sustained directional (downward) movement. As a result, the motion of gravitaxis-deficient worms is primarily governed by passive sedimentation (*F*_p_ ∼ 0 when averaged over the experiment time). Thus, we can estimate the Stokes radius, *r*_s_, from measurements of *cat-2* average settling velocity over time intervals much greater than *τ*_r_.

We monitored worms’ sedimentation across the 5 cm field of view of the cameras to determine their average velocities. *cat-2* mutants (strain MT15620) sedimented at 0.52 ± 0.02 mm s^−1^ (*N* = 30). With eqn (1) and *F*_p_ ∼ 0, we estimate the Stokes radius of 0.37 ± 0.01 mm. The wild-type N2 strain sedimented at 0.64 ± 0.02 mm s^−1^ (*N* = 30). With the aid of eqn (1), we estimate a propulsive force of 0.81 ± 0.14 nN, consistent with prior literature (∼1 nN^18,19^). To our knowledge, the above represents a novel method for measuring the propulsive thrust of worms, utilizing two otherwise similar strains: one taxis-competent and the other taxis-deficient (randomly oriented) under identical cues.

In a second set of experiments, we filled our cuvette with blends of M9 and LUDOX buffers at various compositions. We imaged the behaviors of gravitaxis-competent and gravitaxis-deficient worms for 30 min. We selected a blend with a density of 1.09 g cm^−3^. Wild-type N2 worms released beneath the liquid surface swam deliberately downward. Gravitaxis-deficient worms (MT15620) floated to the top (SI Video S1).

### 3.2 Finite element calculations of the flow field in the sorter

Our numerical simulation revealed that the flow velocity in the feed lines slows down near the junctions with the vertical columns due to the increased effective cross-sectional area. The flow in the horizontal conduits creates vortices in the vertical columns. However, these vortices are too weak to trap worms. The streamlines indicate there is no convection through the vertical columns. The vertical extent of these vortices is in the same order of magnitude as the conduit's width, about 3 mm. The magnitude of the velocity of the circulating flow is about 10^−4^ m s^−1^. The corresponding drag force of 0.3 nN is insufficient to overcome the worm's propulsive thrust and is unlikely to affect the sorter's performance significantly.

### 3.3 Numerically simulated sorting

To estimate our sorter's specificity, we modeled spherical particles with the same Stokes radius (*r*_s_) as *C. elegans* (section 3.1), suspended in the flow field described in section 3.2. The particles were assumed to be passive tracers that do not affect the surrounding fluid's motion. A total of 600 “gravitaxis-competent” particles (blue) and 600 “gravitaxis-deficient” particles (red) were introduced into the computational domain through the bottom feed conduit.

“Gravitaxis-competent” particles had a constant downward propulsive thrust, while the “gravitaxis-deficient” particles experienced thrust vectors randomly reoriented every 4.65 s, mimicking experimental observations of *cat-2* worms. We subjected each particle to a buoyancy force of 0.58 nN and a viscous drag force of 6π*µr*_s_*U*_R_, where *U*_R_ is the relative velocity difference between the particle and the local fluid.

We computed particle trajectories using the Particle Tracer Module in COMSOL, which integrates the equations of motion (eqn (1)) to update particle positions at each time step. A total of 600 “gravitaxis-competent” particles (blue) and 600 “gravitaxis-deficient” particles (red) were introduced into the computational domain through the bottom feed conduit (Fig. 3B–E). The “gravitaxis-deficient” particles exhibited upward drift with erratic paths due to their random propulsion directions. They were ultimately removed through the top collection line (SI Video S2), illustrating that the randomly oriented propulsive thrust does not contribute, over time, to net displacement. In contrast, driven by their consistent downward thrust, the “gravitaxis-competent” particles remained near the bottom of the vertical columns and exited through the bottom feed line. Because all particles remained “motile” throughout the simulation, our sorter achieved 100% specificity in separating “gravitaxis-deficient” from “gravitaxis-competent” particles.

### 3.4 Single-strain worm sorting

We first evaluated our sorter's performance in single strains using day-one adult worms of the following strains: the gravitaxis-competent GFP-expressing strains NQ1155 and PD4790, and the gravitaxis-deficient *cat-2* strain MT15620. Washed and well-fed worms were suspended in the working fluid, loaded into a 10 mL syringe, and introduced into the sorter's bottom feed line at an average fluid velocity of 0.5 mm s^−1^. Given the sorter's internal length (40 mm), it took approximately 80 seconds for a liquid parcel to traverse from the inlet to the outlet. All sorting experiments were concluded within 30 minutes of the worms’ removal from their agar plate and 20 minutes from their introduction into the syringe.

We introduced worms through the bottom inlet rather than the top to minimize sorting time. Gravitaxis-deficient worms, introduced at the bottom, experience upward buoyant force. If gravitaxis-competent worms were introduced from the top, their net downward force would be the difference between their propulsive thrust and the opposing buoyant force, yielding a smaller effective force than buoyancy alone. As a result, they would take longer to reach the bottom than gravitaxis-deficient worms take to reach the top, prolonging the sorting process.

We characterize the sorter's performance using sorting efficiency, defined as the fraction of input worms of a given type that are correctly sorted. When *N*^*C*^_0_ gravitaxis-competent worms are introduced into the sorter, and *N*^*C*^_b_ worms are discharged at the bottom (feed line, true-positives), the efficiency is defined as2
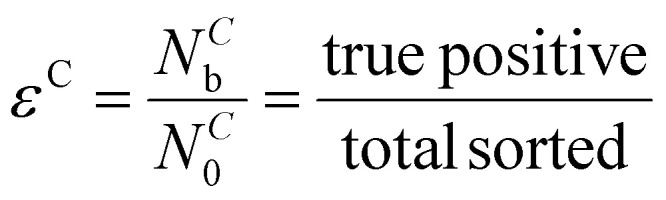


In the above, the superscript “*C*” indicates taxis-competent, subscript “b” bottom, and subscript “0” total (true positives + false negatives). For taxis-deficient worms, when *N*^*D*^_0_ worms are introduced into the sorter and (*N*^*D*^_T_) are discharged at the top, the efficiency is defined as3
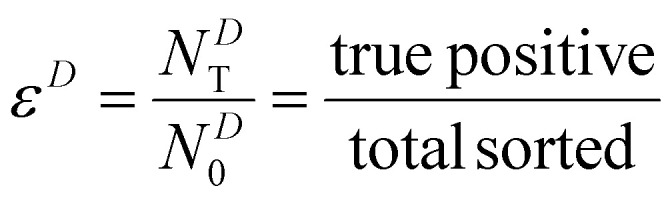


Here, superscript “*D*” indicates taxis-deficient and subscript “T” indicates top.

The gravitaxis-competent strains N2 (*N* = 969), NQ1155 (*N* = 1027), and PD4790 (*N* = 1021) behaved similarly. On average, after 20 minutes of sorting, 88% of N2 (*ε*^*C*^ = 0.88), 89% of NQ1155, and 84.5% of PD4790 remained near the bottom and were discharged through the bottom outlet (SI Tables S2–S4). The remaining worms reached the top collection conduit and discharged into the top collection tube (false positives).

Next, we examined whether the top-collected, gravitaxis-competent worms failed to resist buoyancy due to impaired mobility. To test this hypothesis, we compared the gait frequencies of PD4790 worms collected from the top (*N*_T_ = 150) and bottom (*N*_B_ = 150) outlets. The average gait frequencies of top- and bottom-discharged worms were *f*_T_ = 3.50 ± 0.46 Hz and *f*_B_ = 3.62 ± 0.35 Hz, respectively. A Student's *t*-test yielded a *p*-value of 0.005, indicating a statistically significant difference in gait frequency between these two populations. In contrast, comparisons between groups collected from the same outlet in separate experiments (top *vs.* top and bottom *vs.* bottom) showed no significant differences (*p* > 0.05). The worm's propulsive thrust is proportional to the body undulation's frequency *f*.^20^ Propulsive thrust estimates using both resistive force theory and slender-body theory suggest that worms exhibiting a ∼3% lower gait frequency would still generate enough propulsive forces to overcome buoyancy under our experimental conditions. While this slight reduction in propulsive force may contribute marginally, it is unlikely to be the primary factor underlying the observed false positive rate. Nevertheless, sorting efficiency could be improved by excluding motility-defective worms using a motility-based sorter,^2,21^ either before or after gravitaxis-based sorting.

In contrast, motility is not required to sort gravitaxis-deficient worms. Approximately 94% (*ε*^*D*^ = 0.94) of *cat-2* (MT15620) worms migrated to the top collection line, predominantly due to buoyancy (SI Table S4). A fraction of the gravitaxis-deficient worms remains, however, in the feed line, likely because they have limited time – approximately 6 seconds (3 mm/(0.5 mm s^−1^)) – to interface with and enter any vertical separation column through its bottom. This duration is sufficient for buoyant forces to carry a passive buoyant worm from the bottom to the top of the feed line, but it is too short to fully average out the contribution of the worm's randomly oriented propulsive thrust. A fraction of the gravitaxis-deficient worms may exhibit a swimming velocity component that opposes buoyancy, prolonging the time required to reach the top of the feed line. Additionally, the proximity to the bottom surface induces a hydrodynamic torque that rotates the worms toward the bottom surface, biasing the distribution of orientations. This phenomenon, dubbed bordertaxis,^2,21^ increases the tendency to stay near the bottom.

### 3.5 Sorting efficiency (*ε*^*C*^) declines with time

Our sorter relies on sustained swimming activity of gravitaxis-competent worms during their residence in the device. Prior studies have shown that young adult *C. elegans* can swim continuously for up to 90 minutes without significant pauses,^22,23^ suggesting that the sorter can operate effectively for this duration.

**Fig. 4:**
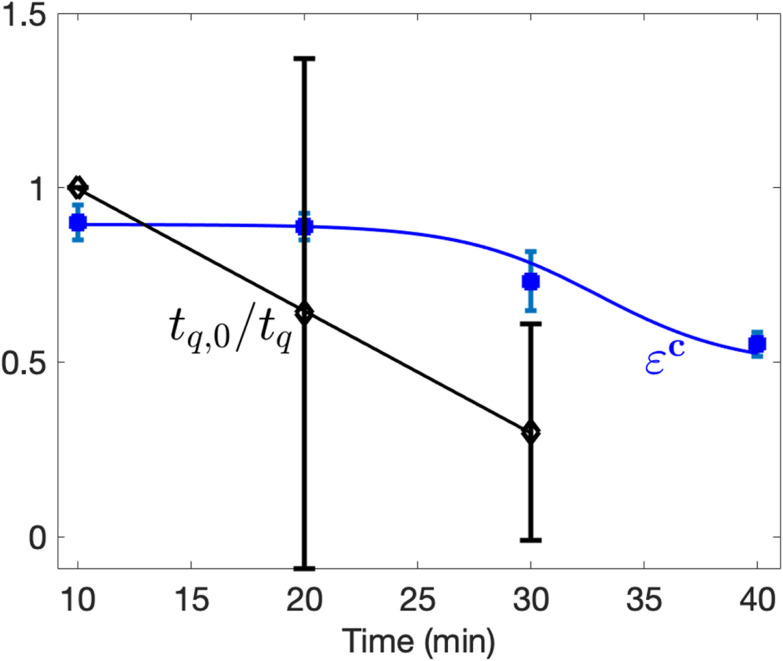
Sorting specificity declines as average quiescence duration increases over time. Fraction of gravitaxis-competent animals (*ε*^C^, blue squares, true negatives) exiting at the bottom outlet and the normalized fraction of average ineffective swimming time intervals (due to quiescent and coil-uncoil behavior) (*t*_q_/*t*_q,0_)^−1^ per animal (black, hollow diamonds, *N* = 304) within 10 min time intervals as functions of time (SI section S6 and Table S13). The sorting efficiency experiment was carried out in triplicate (*N*_1_ = 439, *N*_2_ = 350, *N*_3_ = 483). Error bars represent ± SD.

To evaluate this, we measured the fraction of gravitaxis-competent animals discharged through the bottom outlet (true negatives) as a function of the time elapsed since their removal from the nutrient-rich agar plate. The sorting specificity (blue line, Fig. 4) remained stable for the first 20 minutes after removal but declined sharply thereafter. This decline is well captured by the logistic function (*R*^2^ = 1.0):4
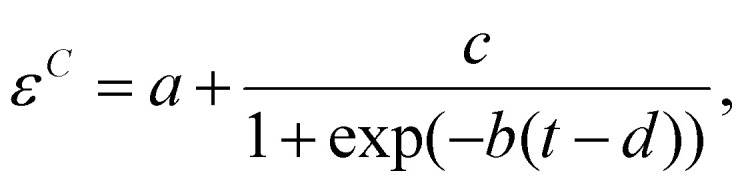
where *t* is time in minutes, and the fitted coefficients are *a* = 0.49, *b* = −3.29 min^−1^, *c* = 0.41, and *d* = 3.3 min. The progressive decline in sorter specificity over time suggests that the worms’ sustained swimming bouts decrease markedly after approximately 30 minutes.

The decline in specificity could be attributed to several factors: (i) increased quiescence after prolonged residence in our device, (ii) oxygen depletion, which has been reported to reduce continuous swimming duration to ∼30 minutes,^22,23^ and (iii) accumulation of toxic metabolic byproducts. Importantly, leachates from the sorter material, if any, do not appear to significantly impact motility (SI section S4). Biological waste, such as carbon dioxide, however, could be contributing to the decline in sorting specificity.

To further examine the effects of prolonged immersion in M9 buffer on mobility, we suspended gravitaxis-competent worms in a 5 mm-deep M9 solution overlaying a bacteria-seeded agar surface. The worms swam nearly parallel to the agar surface. Over time, their gait frequency decreased (SI S5),^23^ while the duration of swimming pauses^22,24–26^ and coil–uncoil intervals, which do not contribute to propulsion, increased^27^ (Fig. 4). Following these pauses and coiling episodes, the worms typically resumed normal swimming behavior. Since our sorting mechanism relies on gravitaxis-competent worms actively swimming against buoyant forces, any reduction in swimming effectiveness leads to reduced sorting specificity (Fig. 4). We suspect that the main cause for reduced sorting specificity with time resulted from increasing quiescent behavior. These findings underscore the importance of limiting sorting duration to less than 30 minutes, with an optimal window of 20 minutes or less after removal from the agar substrate. In contrast, gravitaxis-deficient animals float to the surface due to passive buoyancy forces. Hence, we expect the fraction of false negatives that exit through the bottom port to decline as the sorting time increases.

### 3.6 Pharmacological rescue

*cat-2* mutant worms lack the function of the enzyme tyrosine hydroxylase, which catalyzes the conversion of l-tyrosine to l-3,4-dihydroxyphenylalanine (l-DOPA) − a precursor to dopamine.^28^ As a result, *cat-2* mutants are deficient in dopaminergic neurotransmission. Supplementing these mutants with l-DOPA should, in principle, restore dopamine synthesis.

We added 0.5 mL of a 50 mM l-DOPA solution to the surface of the bacterial lawn on the agar plate. The l-DOPA dissolved into the M9 buffer and was partially absorbed by the agar, with the highest concentration remaining near the surface. l-DOPA readily oxidizes in air,^29,30^ producing toxic byproducts.^31–33^ To minimize the accumulation of these oxidative products, we transferred worms to freshly prepared l-DOPA plates every 48 hours, as previously recommended.^29^ In another set of experiments, we cultured *cat-2* worms to adulthood without l-DOPA and then administered l-DOPA for 1 hour to one-day-old adults, demonstrating that the rescue is acute.

We added the l-DOPA-treated animals to our sorter. Most treated *cat-2* worms (76%, *N* = 1125) swam downward, indicating restored gravitaxis and confirming their ability to sense and respond to gravity (SI Table S6 and Video S1). l-DOPA is highly unlikely to acutely affect the worms’ distribution of mass; thus, this pharmacological rescue result further supports the notion that gravitaxis behavior is not caused by top heaviness of the worms.^6^

To mitigate l-DOPA oxidation, we supplemented the solution with 1 mM ascorbic acid, an antioxidant known to stabilize l-DOPA.^29^ In a separate control group, we also treated *cat-2* mutants with 1 mM ascorbic acid alone (without l-DOPA) to isolate its effect. After 72 hours of treatment, we sorted one-day-old adult worms. Among those treated with l-DOPA and ascorbic acid (*N* = 1124), nearly 89% remained at the bottom of the sorter (SI Table S7), showing a greater gravitaxis restoration than with l-DOPA alone. This enhancement is likely due to reduced oxidative stress and increased l-DOPA bioavailability in the presence of ascorbic acid. In contrast, ascorbic acid alone failed to rescue the gravitaxis phenotype – only 12.3% of the treated worms (*N* = 1067) remained at the column's bottom (SI Table S8).

These results confirm that l-DOPA can pharmacologically rescue the gravitaxis defect caused by cat-2 gene knockout, supporting the role of dopamine in the gravity-sensing circuitry. Furthermore, this experiment highlights the potential of our sorter for evaluating pharmacological efficacy.

### 3.7 Sorting specificity

Our gravitaxis sorter is designed to isolate gravitaxis-deficient animals from a mixed population containing mostly gravitaxis-competent worms and rare gravitaxis-deficient worms. We define sorter specificity (*S*_p_) as the ratio of gravitaxis-deficient worms (true positives, collected at the top) to the total number of worms (true positives + false positives) collected at the top outlet.5
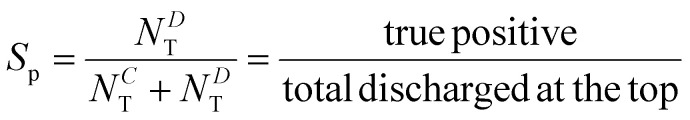


Specificity (*S*_p_) may decline with increasing worm residence time in the syringe pump and sorter (Fig. 4).

Sorted worms were counted on an agar plate using a stereomicroscope under bright-field illumination. Since gravitaxis-competent and -deficient worms are visually indistinguishable under these conditions, we used the GFP-expressing, gravitaxis-competent fluorescent strain PD4790 and the non-fluorescent, gravitaxis-deficient strain *cat-2* (MT15620) (SI section S9). A mixed population of PD4790 (*N* = 674) and *cat-2* (MT15620, *N* = 736) worms was sorted for 30 minutes after removal from their agar lawns.

Following sorting, the upper collection tube contained 85% (*S*_p_ = 0.85) non-fluorescent, gravitaxis-deficient (*cat-2*) worms (true positives) and 15% fluorescent, gravitaxis-competent (PD4790) worms (false positives). The lower collection tube contained 86% fluorescent worms (true negatives) and 14% non-fluorescent worms (false negatives) (SI Table S11). These results are consistent with those obtained using an alternative fluorescent, gravitaxis-competent strain (SI section S7) and with those presented in sections 3.4 and 3.5. A 30 minute sorting run yields a specificity of approximately 84%.

#### Repeated sorting improves specificity

In a forward genetic screen, worms collected from the top outlet of our sorter will ultimately undergo resource-intensive DNA sequencing and genetic analysis. After one sorting round, ∼10% of N2 worms were collected at the top of the sorter, *i.e.*, they were falsely classified as gravitaxis defective.

To minimize the number of gravitaxis-competent false positive worms pursued in future molecular genetic work, it is desired to maximize sorter specificity. Refilling the top-sorted worms directly into the sorter is not viable, as specificity declines with increasing residence time in our device. Instead, we placed the top-collected worms on an agar plate and allowed them to feed for 2 hours, thereby restoring their original activity levels before re-sorting.

**Fig. 5:**
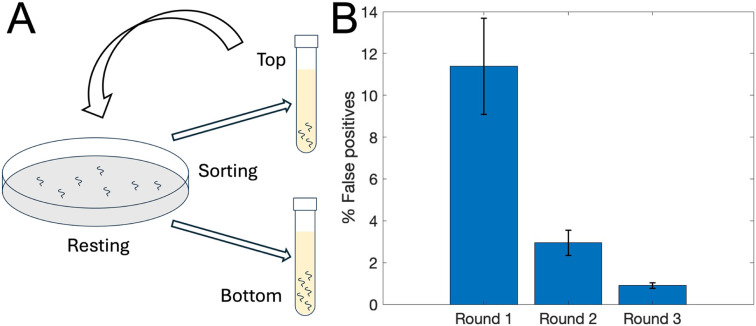
(A) Repeated sorting improves specificity. (B) Aggregate percentage of false positives as a function of the number of sorting rounds. Experiments were repeated three times, starting with 462, 388, and 204 N2 worms in each run, respectively. Error bars denote standard deviation.

We repeated the sorting of N2 gravitaxis-competent worms twice more (Fig. 5). After each round, worms collected from the top outlet were transferred to M9-saturated agar plates seeded with bacteria. Following a 2 hour recovery period, we subjected these rested worms to another sorting round. After three sorting rounds, the fraction of false positives (gravitaxis-competent worms incorrectly collected from the top outlet) decreased to ∼1% (Fig. 5). Correspondingly, the cumulative fraction of worms collected from the bottom outlet across all rounds reached 99.10 ± 0.25% of the total population (Table S14). This sequential sorting strategy enhances sorting efficiency. The availability of data on sorting specificity, *ε*_*k*_^*C*^, at each sorting round *k* allows an experimenter to plan the number of sorting rounds needed to achieve the desired efficiency. For example, after *K* sorting rounds, the cumulative sorting efficiency of gravitaxis-competent worms is: 
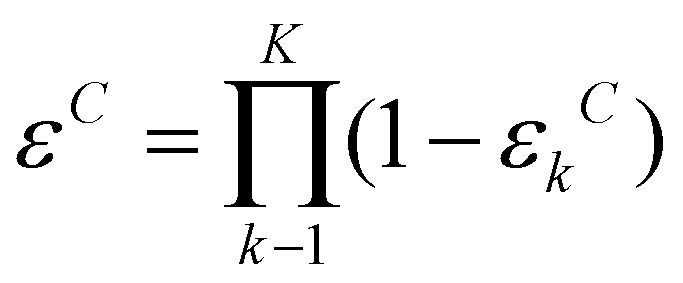
. Increased sorting efficiency of taxis-competent worms increases specificity.

## 4. Discussion

A major strength of the *C. elegans* model is its suitability for phenotype-driven forward genetic screens, in which mutants displaying defects in a defined biological process are isolated and the causative genes subsequently identified. These screens are most effective when conducted in a high-throughput format to recover rare mutants, which can then undergo molecular genetic analysis to uncover genes governing the behavior of interest. Characterizing the expression patterns and functions of these genes provides insight into the underlying neural circuits and can inform therapeutic discovery. Despite the advantages of *C. elegans*, identifying genes responsible for taxis defects remains resource-intensive, requiring genetic mapping, genome sequencing, and transgenic rescue. Minimizing the misclassification of taxis-competent animals as taxis-deficient (false positives) is therefore essential to reduce the downstream workload.

A longstanding bottleneck in stimulus–response genetics has been the lack of efficient, continuous methods to isolate taxis-deficient individuals from large populations of taxis-competent worms. To address this need, we propose a microfluidic sorting platform that separates worms based on functional taxis behavior. Our device exploits the balance between stimulus-driven locomotory thrust and an externally applied, finely tuned passive counterforce that transports worms in the opposite direction. The nature of the counterforce can be tailored to the sensory modality under investigation. For example, buoyancy (gravitational pull) opposes vertical displacement in positive (negative) gravitaxis assays, or a precisely controlled adverse flow opposes directed propulsion in chemotaxis or thermotaxis assays. Worms with intact taxis generate sufficient propulsive force to overcome the imposed adverse force and migrate toward the stimulus, whereas taxis-deficient individuals fail to counteract this adverse force and are displaced in the opposite direction. This principle enables high-throughput, phenotype-specific enrichment of behaviorally defective worms, facilitating iterative selection to increase stringency and enabling downstream genetic analysis.

The high-throughput capacity of GravSorter and the ease with which sorted animals are collected enable unbiased forward genetic screens in which mutagenized worms are phenotypically sorted according to taxis competence. GravSorter's selection mechanism depends on animals generating sufficient propulsive thrust to overcome buoyant force and sustain directed downward swimming. Consequently, worms with primary locomotory impairments may be misclassified as gravitaxis-deficient, even if their gravity sensing and neural processing are intact, thereby reducing sorter specificity.

To mitigate this confounding factor, animals generating propulsive thrust below a defined threshold (*e.g.*, less than the effective buoyant force) should be excluded prior to gravitaxis sorting. This can be accomplished using a previously described tunable motility sorter,^2^ which removes low-thrust worms from the population before they enter GravSorter. Integration of this module upstream of the GravSorter feed line provides a streamlined prescreening step, functionally decoupling locomotor capacity from sensory-driven orientation. As a result, downstream enrichment will more accurately reflect taxis-specific phenotypes rather than nonspecific defects in motility.

GravSorter performance also depends on the worm–medium density differential, which determines the magnitude of buoyant force. This factor is deemed less critical than propulsive thrust because reported body density in age-matched *C. elegans* populations varies within a relatively narrow physiological range.^16^ Within this range, buoyant force differences are small compared with the thrust generated by actively swimming worms and thus are unlikely to dominate sorting outcomes under properly tuned conditions. If density variation becomes non-negligible, for example, in populations with altered lipid content, developmental stage differences, or metabolic mutants, the buoyant offset can be tuned by adjusting the density of the M9/LUDOX medium to maintain the desired force balance. Additionally, a continuous-flow density-based pre-sorting module could be implemented upstream to normalize the population for body density before behavioral sorting.

Maximizing sorter specificity requires that gravitaxis-competent animals sustain continuous propulsive activity throughout the sorting interval. Episodic quiescence introduces false positives because transiently inactive, yet sensory-competent, worms lose thrust and are swept by buoyant force, leading to passive upward displacement. We observe stable sorting performance for ∼20 minutes, followed by a rapid decline (Fig. 4), indicating that operational duration should be limited to ≤20 minutes. We attribute this deterioration primarily to activity-state transitions rather than sensory failure.

Although young adult worms have been reported to sustain prolonged swimming (>30–60 min) under conventional assay conditions,^26,27^ locomotory endurance in the GravSorter appears reduced. Several mechanistic factors may contribute. First, sustained swimming in confined columns likely accelerates neuromuscular fatigue, including depletion of readily available ATP, accumulation of metabolic byproducts (*e.g.*, carbon dioxide and lactate), and reduced calcium handling efficiency in body-wall muscle. Second, limited fluid exchange may reduce local oxygen availability, increasing reliance on less energy-efficient anaerobic metabolism. Third, mechanosensory and surface-contact signaling in confined geometries may promote transitions to quiescent states, which in *C. elegans* are regulated by neuro-modulatory pathways linked to energy balance and stress.^34^ Finally, exposure to trace leachates from partially cured 3D-printed materials used to fabricate the sorter may impose low-level toxic stress, thereby diminishing locomotor persistence and endurance.

In forward genetic screens, the principal bottlenecks are downstream sequencing, genetic crosses, and data analysis; therefore, minimizing false positives during the sorting stage is a priority. To reduce false positives arising from activity-state transitions, the residence time within the GravSorter can be limited to remain within the worms’ high-activity window, thereby reducing fatigue and stress-induced behavioral variability. Furthermore, worms can be allowed to recover with feeding between sorting rounds to restore metabolic state and neuromuscular performance, improving phenotype stability and classification specificity in subsequent passes. Consistent with this strategy, we have demonstrated that repeated sorting substantially enhances overall sorter efficiency and enriches true phenotype-positive populations.

Because mutagenized populations are expected to contain multiple independent alleles at each genetic locus, we prioritize minimizing false positives over false negatives to reduce the downstream burden of mapping and sequencing. False positives and false negatives arise from distinct physical mechanisms intrinsic to the sorter's operation. False positives primarily result from episodic quiescence. Gravitaxis-competent worms that transiently cease swimming fail to generate propulsive thrust sufficient to offset buoyancy. In the absence of active downward swimming, they are displaced by the passive counterforce and misclassified as gravitaxis-deficient. False negatives, in contrast, arise from active behavior during entry into the sorting columns. To be correctly classified, gravitaxis-deficient worms must transition from the feed line into the vertical columns. These animals are motile, randomly oriented, and hydrodynamically biased toward nearby surfaces (bordertaxis).^3,21^ Consequently, a subset remains in the vicinity of channel boundaries and is advected through the feedline toward the outlet, where it is discharged despite lacking directional gravitactic bias. Hence, transient inactivity inflates false positives, whereas sustained activity combined with wall-mediated hydrodynamic interactions contributes to false negatives.

In summary, we have developed a microfluidic platform that separates *C. elegans* based on gravitaxis. The device operates in a working fluid slightly denser than the worms, so buoyancy provides a passive upward force that opposes active downward swimming. Gravitaxis-deficient worms, though motile, lack a sustained downward bias and therefore rise with buoyancy to the upper collection conduit. In contrast, gravitaxis-competent worms deliberately swim downward, generating sufficient thrust to counter buoyancy, and remain in the lower conduit, where they are discharged to waste. This force-balance mechanism enables efficient, high-throughput enrichment of gravitaxis-deficient animals.

We established the operating principle by numerical simulation and validated performance experimentally using gravitaxis-competent strains (N2, GFP-expressing NQ1155 and PD4790) and the gravitaxis-deficient mutant *cat-2* (MT15620). The sorter achieved robust separation in both pure and mixed populations, with >85% specificity after a single round and >99% specificity after three sequential sorting rounds.

Finally, we demonstrated pharmacologic rescue of gravitaxis in *cat-2* mutants by exogenous dopamine supplementation. This capability extends the utility of GravSorter beyond genetic screening to quantitative assessment of drug efficacy (*via* responder fraction) and to the identification of mutations that confer resistance to therapeutic intervention.

Recently, Ackley *et al.*^35^ reported negative gravitaxis in wild-type dauer and adult *C. elegans* crawling within a narrow gap between a vertical 4% agar surface and a pipette wall, a finding that contrasts with the positive gravitaxis observed in our study. Several differences may account for these apparent divergent polarities: (A) Physical environment. Ackley *et al.* examined worms crawling on agar, whereas our experiments (ref. 6 and this study) involve animals in liquid, a distinction that fundamentally alters both sensory and mechanical inputs. (B) Physiological state. Ackley *et al.* focused on dauer larvae and on adults deprived of nutrition; our work examines well-fed, first-day adults. Developmental stage and nutritional status profoundly affect neuromodulatory tone and metabolic state, factors that could shape gravity-dependent behavioral responses. (C) Timescale. Ackley *et al.* quantified behavior after 12–24 h of confinement in a sealed vertical tube, allowing time for adaptation and behavioral state transitions. Prolonged confinement in such a geometry can generate internal gradients of O_2_, CO_2_, and other metabolites and may also deform the tall agarose slab, altering the gap thickness and thereby biasing locomotion. In contrast, our GravSorter operates in liquid with freely swimming animals over minute timescales. Differences in locomotion mode, physiological state, and assay duration therefore provide plausible explanations for the opposite gravitaxis polarity observed in the two studies.

Ackley *et al.*^35^ reported that environmental electromagnetic (EM) waves and light can confound worm behavior by interfering with sensory processing. To test whether these factors influenced our gravitaxis assays, we introduced N2 worms into a LUDOX-filled vertical column with a density closely matching that of the worms (1.08 g mL^−1^). The column was encased in a black, opaque cloth and aluminum foil shield (Faraday cage) for 10 min, and the worms’ distribution was imaged immediately after the shields were removed. As in our previous experiments, most worms accumulated at the bottom of the column, demonstrating positive gravitaxis. This persistence of positive gravitaxis in a density-matched, shielded environment confirms that the observed behavior is a primary response to gravity rather than a secondary response to environmental artifacts.

Future work will leverage the GravSorter as a high-throughput platform to identify additional genes and neural circuit elements required for gravitaxis. By combining forward genetic screens, targeted knockdowns, and pharmacologic rescue experiments, we aim to map the sensory neurons, interneurons, and effector pathways that mediate gravity-response in *C. elegans*. Since many mechanosensory, proprioceptive, and neuromodulatory pathways are evolutionarily conserved, candidate genes and circuit motifs identified in worms can be cross-referenced with homologous pathways in more complex organisms, including humans. This comparative approach may reveal conserved molecular components of balance and orientation control.

Impairments in balance and postural regulation represent a major and growing public health concern: falls are a leading cause of injury, morbidity, and mortality among the elderly and people with neurological impairments. A genetically tractable model that permits scalable genotype–phenotype mapping of gravity-related behaviors provides an opportunity to dissect fundamental mechanisms of orientation sensing and motor coordination, and to explore pharmacologic or genetic modifiers that restore function. In this context, GravSorter offers a quantitative, mechanistically grounded framework for linking genes, neural circuits, and balance-related behavior across species.

We identified dopamine as a key modulator of gravitaxis in *C. elegans*. This finding is mechanistically plausible given dopamine's broad functional roles in the worm, including regulation of mechanosensation associated with bacterial encounters,^36^ modulation of locomotory speed and precision,^37^ control of growth^38^ and developmental timing, and activation of the mitochondrial unfolded protein response.^39^ These diverse functions suggest that dopamine may influence gravitaxis either directly, through modulation of sensory or interneuronal circuitry, or indirectly, by altering neuromuscular performance, endurance, or metabolic state. Future work should dissect whether dopamine acts at the level of gravity sensing, sensorimotor integration, and/or motor execution (Fig. 1).

The relevance of dopaminergic signaling to gravity-related behavior is further supported by studies conducted under altered-gravity conditions. Notably, Sudevan *et al.*^40^ demonstrated that *C. elegans* under microgravity exhibited impaired dopamine production, reduced motor activity, and weight loss, and exogenous dopamine reversed these physiological deficits. These findings, consistent with ours, underscore dopamine's central role in maintaining motor competence and adaptive behavioral responses under gravitational challenge.

Because dopaminergic neuron degeneration in *C. elegans* is widely used as a model of neurodegeneration,^41,42^ the GravSorter provides a quantitative, scalable platform for detecting early functional impairment through behavioral phenotyping before overt neuronal loss occurs. Its efficiency and reproducibility position it as a tool not only for dissecting the gravitaxis circuitry but also for identifying genetic and pharmacologic modifiers of dopaminergic dysfunction.

The identification of a dopamine-deficient strain as gravitaxis-impaired raises broader questions regarding the role of neuromodulation in balance control. Falls among older adults and individuals with neurodegenerative disorders, particularly Parkinson's disease (PD), represent a major clinical problem associated with significant morbidity, mortality, and healthcare burden. The neural and molecular mechanisms underlying gravity perception and postural regulation remain incompletely defined. Greater insight into the circuit- and gene-level determinants of gravity-directed behavior may therefore contribute to a deeper understanding of balance dysfunction and its progression in disease. Although direct extrapolation from *C. elegans* to humans is not implied, it is notable that recurrent fallers among PD patients exhibit reduced striatal dopamine levels,^42^ consistent with an important role for dopaminergic signaling in balance-related motor control.

## Conflicts of interest

There are no conflicts to declare.

## Supplementary Material

AN-151-D5AN01231G-s001

AN-151-D5AN01231G-s002

AN-151-D5AN01231G-s003

## Data Availability

All data is available in the article's supplementary information (SI). Supplementary information is available. Including representative videos of forward and reverse genetic screens (N2, *cat-2*, and rescued *cat-2* strains) and tables summarizing sorting performance. See DOI: https://doi.org/10.1039/d5an01231g.
