# Supplementary material for: GravSorter: a forward-genetics tool for studying gravity response in Caenorhabditis elegans
Source: Analyst. 2026 Apr 20;151(11):3118–29. doi: 10.1039/d5an01231g (PMC13123466; doi:10.1039/d5an01231g)
Supplement: AN-151-D5AN01231G-s001 [file AN-151-D5AN01231G-s001.pdf]

Supplementary Information (SI) for Analyst.
This journal is © The Royal Society of Chemistry 2026

Supplemental Information

GravSorter: A Forward-Genetics Tool for Studying Gravity Response in *Caenorhabditis elegans*

Hui Ma[1], David M. Raizen[2], and Haim H. Bau[1]

[1]Department of Mechanical Engineering and Applied Mechanics, University of Pennsylvania, Philadelphia, PA, USA

[2]Department of Neurology, Perelman School of Medicine, University of Pennsylvania, Philadelphia, PA USA

Videos

Video S1: GravSorter: Reverse and Forward Genetic Screens.

Reverse Screen: Worms are suspended in a liquid column less dense than the worms. Both gravitaxis (g)-competent and g-deficient worms sink to the bottom. Gravitaxis-competent worms deliberately align their swimming direction downward, augmenting gravitational pull with propulsive thrust. In contrast, g-deficient worms, such as *cat-2*, orient randomly in space, so their propulsive thrust does not contribute to their downward velocity over time. This assay enables the assessment of whether a given strain responds to gravity.

Forward Screen: An assortment of randomly mutated worms is suspended in a medium slightly denser than the worms. Gravitaxis-competent worms deliberately swim downward, overcoming buoyancy, and are discharged to waste. Gravitaxis-deficient worms float to the top and are collected for further sorting and analysis.

Pharmaceutical Rescue: Worms unable to process gravitational information due to defects in dopamine synthesis, such as *cat-2*, can be rescued with exogenous dopamine. Our assay also enables testing of drug efficacy.

Video S2: Animation of the sorter's operation. Gravitaxis-competent active particles (blue) with downward-directed propulsive thrust and gravitaxis-deficient particles (red) with randomly re-orienting propulsive thrust in a vertical cuvette filled with LUDOX buffer that is 1% denser than the particles. The particles have the same Stokes radius as the worms. The flow field and particles' trajectories were computed with the Multiphysics, finite-element program COMSOL version 6.2. Gravitaxis-competent particles defy buoyancy and exit through the bottom line. In contrast, gravitaxis-deficient particles float to the top and exit through the top collection line.

Table of Contents*

S1. Confirmation of *cat-2* gene deletion in the MT15620 strain 3

S2. Propulsive Thrust Measurement Methods 3

S3. Factors Affecting Sorting Efficiency 4

S4. Resin Toxicity 6

* Section, figure, table, and reference numbers preceded by the letter "S" refer to items contained within this Supplementary Information. Numbers without the "S" prefix refer to corresponding items in the main manuscript.

S5. Sorting Efficiency 6

S6. Quiescence is the likely cause of less-than-perfect efficiency 7

S7. Pharmacological Rescue 8

S8. Fluorescent Ratio of NQ1155 8

S9. Sorter Specificity 9

Supplemental References 12

List of Figures

S1. Confirmation of *cat-2* gene deletion (gel electrophoresis) 3

S2. Comparison of N2 and *cat-2* body sizes 4

S3. Gait frequency and amplitude of N2 worms collected at the top (false positives) and the bottom (true positives)...5

S4. Gait frequency and amplitude of N2 worms collected at the top (false positives) and the bottom (true positives)...5

S5. Fatigue effect on the worm's gait and quiescence 7

S6. Stereomicroscopy images of mixed populations of sorted *NQ1155/MT15620* worms. 8

List of Tables

S1. Propulsive Thrust in an unbound aqueous medium 4

S2. N2 Sorting Efficiency 9

S3. NQ1155 Sorting Efficiency 9

S4. PD4790 Sorting Efficiency 10

S5. MT15620 Sorting Efficiency 10

S6. N2 Sorting Efficiency as a Function of Sorting Time 10

S7. Sorting efficiency of MT15620 following pharmaceutical rescue with L-DOPA....... 10

S8. Sorting efficiency of MT15620 following pharmaceutical rescue with L-DOPA and ascorbic acid 10

S9. Sorting efficiency of MT15620 following pharmaceutical rescue with ascorbic acid (control) 11

S10. Fluorescence ratio 11

S11. Sorting specificity of a mixed population of MT15620 and NQ1155....... 11

S12. Sorting specificity of a mixed population of MT15620 and PD4790 12

S13. Quiescence duration in swimming N2 adults (Day 1, N=304) across 10-minute intervals 12

S14. The fraction of N2 non-productive swimming intervals as a function of sorting cycle 12

S1. Confirmation of *cat-2* gene deletion in the MT15620 strain.

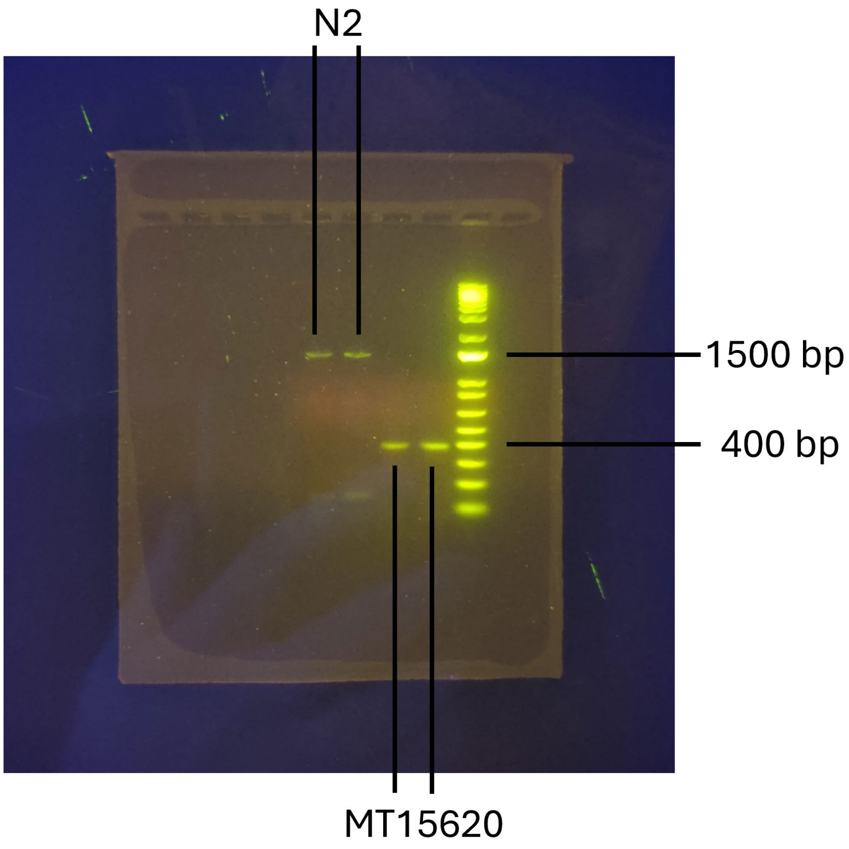

Figure S1: **Confirmation of cat-2 deletion**. Gel electrophoresis of N2 and MT15620 amplicons. The right lane is the DNA ladder. We indicated the locations of 1500 base pairs and 400 base pairs in the ladder. The N2 strain is gravitaxis-able, with a complete *cat-2* gene. The MT15620 strain's cat-2 gene is knocked down, resulting in a shorter PCR amplicon.

Genomic DNA from wild-type and *cat-2* mutant (MT15620 strain) worms were used as templates for PCR amplification across the *cat-2* locus. The deletion was verified by comparing the amplicons' lengths. The PCR forward and reverse primers (Integrated DNA Technologies) were, respectively, **GTCCGTCTTGAGAATCTGATCTTCAT** and **TATGTTTTTGAACTTTAATGGCTTCTATTTCG**. Polymerase (Phusion high-fidelity DNA polymerase (HF buffer), Biolabs) and PCR protocol were from Biolabs and conducted on a thermal cycler (Bio-Rad, C1000 Touch). The DNA templates used in the PCR were DNA genomes of the MT15620 and N2 strains.

Following the PCR reaction, the amplicons were analyzed using gel electrophoresis (Ultrapure Agarose, catalog number 16500-500, Invitrogen) along with a DNA ladder (1 Kb Plus DNA ladder, catalog number 10787018, Invitrogen) to determine their lengths. The gel electrophoresis results showed that the amplicon of the wild-type N2 strain (gravitaxis-able) was longer than that of the MT15620 strain (gravitaxis-deficient). This difference is due to the knockout of approximately 1100 base pairs in the *cat-2* gene of MT15620. As a result, the MT15620 strain cannot synthesize tyrosine hydroxylase, an essential enzyme for converting tyrosine to L-DOPA.

S2. Propulsive Thrust Measurement Methods

Our method for estimating propulsive thrust requires that the gravitaxis-competent N2 and gravitaxis-deficient cat-2 strains have nearly identical body surface area, length, and volume. We imaged both strains using a 5× objective (Fig.

S2) and extracted body contour lengths and cross-sectional areas of N2 (*N=74*) and cat-2 (*N=72*) worms. Consistent with previous reports (S1), *cat-2* mutants were, on average, 6.7% longer than wild-type worms, a difference that was statistically significant (p = 0.019), whereas cross-sectional area did not differ significantly (p = 0.80), which means that cat-2 worms were slimmer than their N2 counterparts. These modest differences in length and radius, although statistically significant, are unlikely to affect our estimate of N2 propulsive thrust (Manuscript Section 3.1). To further verify that our estimate of the propulsive thrust is reasonable, we compared our estimates with experimentally measured and theoretically predicted thrust values reported by other researchers (**Table S1**). The agreement between our results and published data supports the validity of our estimation approach.

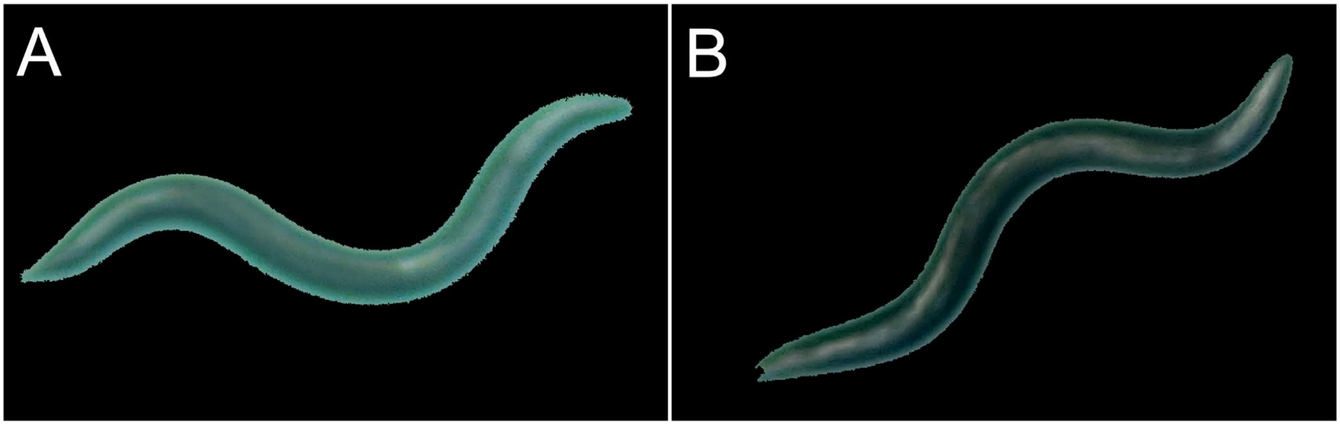

Figure S2: Comparison of N2 (A) and *cat-2* (B) body sizes.

Table S1: **Propulsive Thrust in an Unbound Aqueous Medium**

Propulsive Thrust (nN)	Method	Reference
0.81 ± 0.14	Compare downward velocities of gravitaxis-competent and -deficient worms	**This paper**
0.8 ± 0.2	The worm is tethered to a pulled capillary's tip. The force is estimated from the capillary tip's deflection*.	Schulman et al. [S3]
1.0 ± 0.8	Particle Image Velocimetry (PIV) was employed to analyze the deformation and velocity of the liquid-air interface of a slug confined between two parallel plates that contained a live worm.	Kuo, Sie, and Chuang [S4]
< 1.8	Linear Resistive Force Theory **	Gray and Hancock [S5]

***** Fig. 4 in Schulman et al. [S3].

****** Linear Resistive Force Theory (RFT) overestimates the propulsive force as it does not account for the relatively large amplitude of the undulatory gait when the worm swims in a low viscosity medium (M9 buffer) and neglects hydrodynamic interactions among body length segments.

S3. Factors Affecting Sorting Efficiency

We measured the gait frequency and amplitude of the PD4790 strain. After 20 min of sorting, 30 worms were collected from each of the top and bottom outlets. To minimize starvation effects, these worms were promptly

transferred to an agar plate seeded with bacteria. A thin layer of 3 mL M9 buffer, 5 mm deep, was added to the agar surface, allowing the worms to swim freely. Each worm's gait frequency and amplitude were measured five times.

The gait frequency, defined as the inverse of the gait period (the time required to complete a full undulatory cycle (including both ventral and dorsal bends), and the amplitude, defined as the maximum perpendicular displacement from the worm's centerline to the straight line connecting the head and tail, during a ventral or dorsal bend, showed only minor differences between worms collected from the top and bottom outlets (Fig. S3). Student's t-tests yielded p-values of 0.0051 for gait frequency and 0.0001 for amplitude. Although these differences are statistically significant, they are too small to account for why some PD4790 worms were recovered from the top outlet.

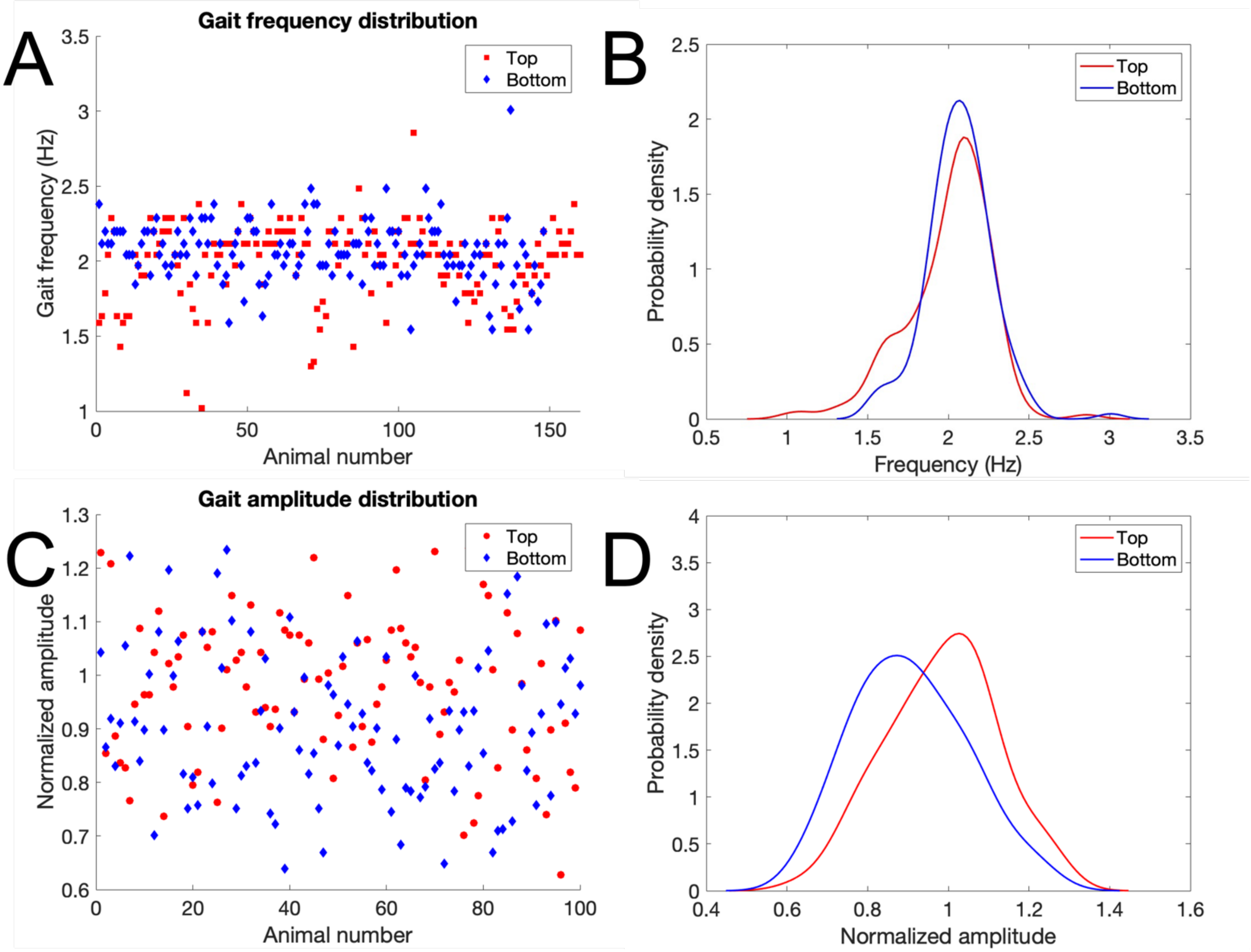

Figure S3. Gait frequency and amplitude of N2 worms collected from the top (false positives) and bottom (true negatives) outlets. Gait frequency (**A**) and normalized amplitude (**B**) are shown for individual worms collected from the top (false positives, red circles, N = 150) and bottom (true negatives, blue diamonds, N = 150). Kernel density estimates (KDE) of gait frequency (**C**) and mean-normalized amplitude (**D**) are also shown for top-collected (red, false positives) and bottom-collected (blue, true negatives) populations. Although the distributions are statistically distinct, the differences are too small to account for the observed false positives.

S4. Resin Toxicity

We tested whether any solvents leaching from the resin used to 3D-print our sorter might adversely affect worm behavior. The sorter was soaked in M9 buffer for approximately 20 hours. A batch of first-day adult wild-type worms was washed and divided into two groups. One group was suspended in the resin-soaked M9 buffer, while the other (control group) was suspended in a fresh M9 buffer. After 20 minutes, we recorded videos and analyzed the worms' gait frequency and amplitude.

The average gait frequencies for the resin-exposed and control groups were 2.4 Hz and 2.3 Hz, respectively, with a p-value of 0.0017 (Fig. S4 A, B). The gait amplitudes have a p-value of 0.0052 (Fig. S4 C, D). Although statistically significant, these small differences are unlikely to account for the failure of wild-type worms to reach the bottom outlet in the sorter.

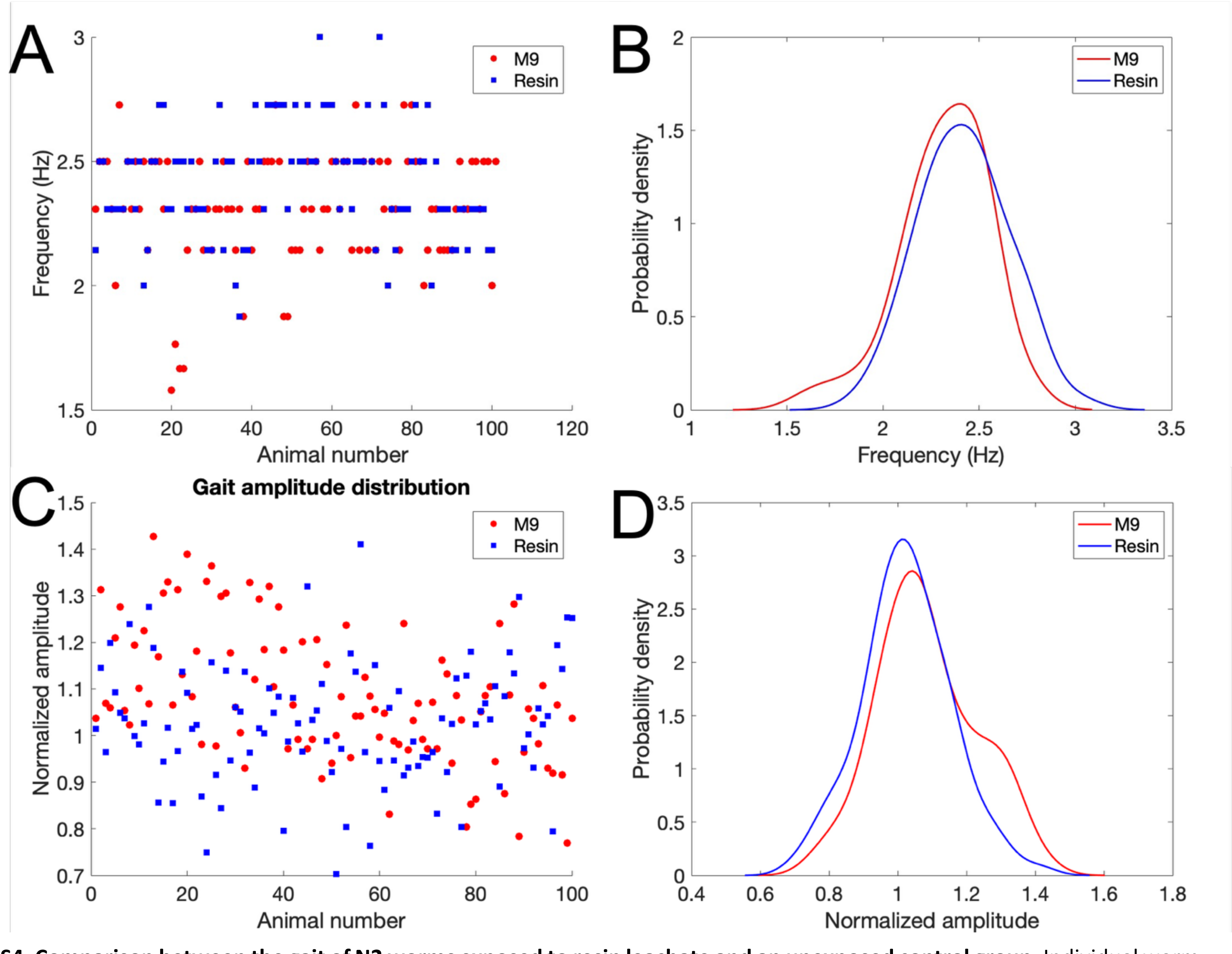

Figure S4: Comparison between the gait of N2 worms exposed to resin leachate and an unexposed control group. Individual worm frequencies (**A**) and mean - normalized gait amplitudes (**C**) following exposure to resin leachate (blue squares, *N* = 100) or leachate-free control (red circles, *N* = 100). Kernel density estimates (KDE) of gait frequency (**B**) and normalized amplitude (**D**) are shown for worms exposed to resin leachate (blue) and control conditions (red).

S5. Sorting Efficiency

We tested each strain independently. The sorter efficiency for gravitaxis-competent animals is defined as $\varepsilon^C = N_B^C/N_0^C$, where N_0^C is the total number of taxis-competent animals introduced into the sorter, and N_B^C is the number of competent animals collected at the bottom outlet. The sorting efficiency for gravitaxis-deficient animals is defined as $\varepsilon^D = N_T^D/N_0^D$, where N_0^D is the total number of taxis-deficient animals introduced into the sorter, and N_B^D is the number of taxis-deficient animals discharged at the bottom.

Worms were introduced into the sorter within 10 minutes of removal from the culture agar plate. Tables S2–S5 report the numbers of worms collected at the top and bottom outlets within 30 minutes of removal from the agar plate and 72 hours post-hatching for the N2, NQ1155, PD4790, and MT15620 strains.

S6. Quiescence is the Likely Cause of Less-than-Perfect Efficiency

Our sorting relies critically on gravitaxis-competent worms maintaining nearly continuous swimming activity. Quiescent or ineffective swimmers float to the top, enter the collection line, and are mistakenly classified as gravitaxis-incompetent. To assess the effect of fatigue on worm behavior, we suspended one-day-old adult N2 worms in a 5-mm-deep M9 buffer above an agar surface (in the absence of bacteria) and monitored their behavior for 1 hour. With time, quiescent intermissions and periods of ineffective swimming (Fig. S5B) increased in duration ($N = 304$, Manuscript Fig. 4 and Table S13), while gait frequency decreased (Fig. S5A). The gait amplitudes at 0 and 40 min do not differ significantly ($p = 0.45$). Since gait frequency correlates with propulsive thrust, this decline indicates reduced ability to counteract buoyancy with increased residence time. After 15, 30, 45, and 60 min, the average worm's propulsive thrust, respectively, declined by 7.5%, 20.5%, 21.0% and 34.0% compared to the propulsive thrust at time zero. Ineffective swimming periods often included coiling and uncoiling (Fig. S5B).

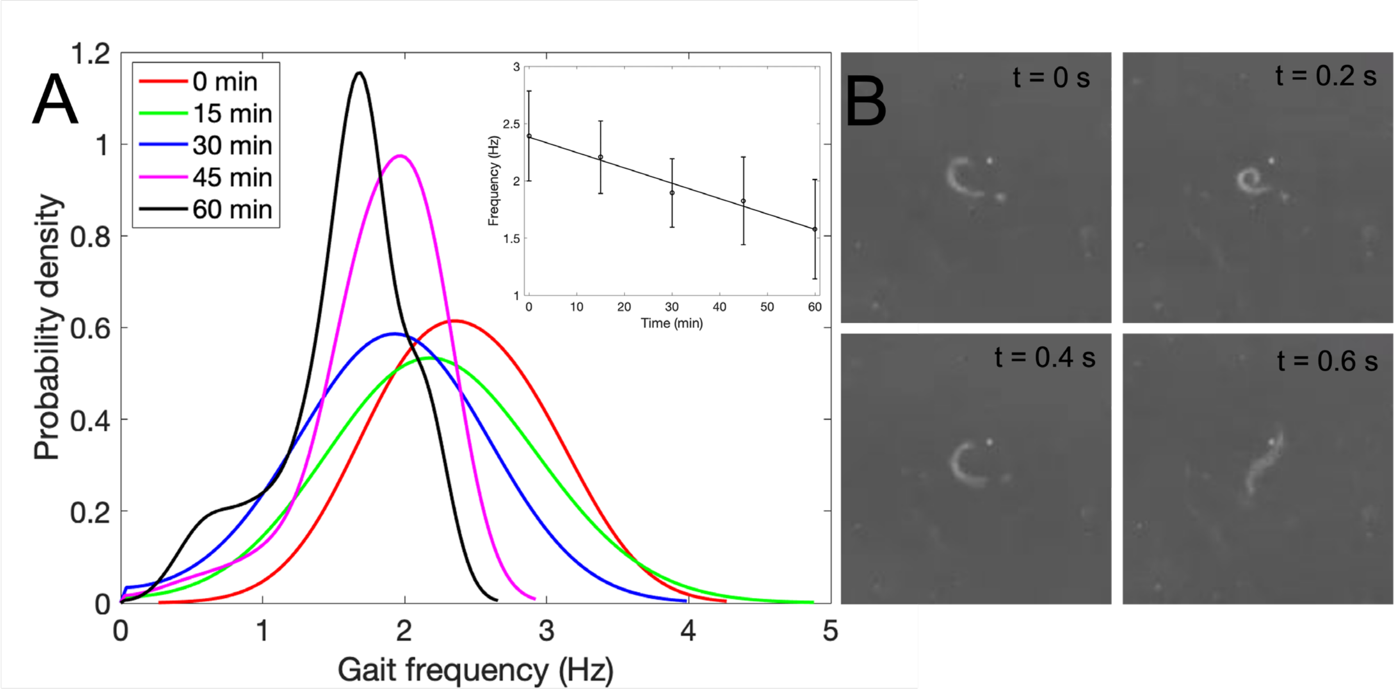

Figure S5: **Fatigue effect on the worm's gait and quiescence.** (**A**.) Worms' gait frequency (N = 100) KDEs after 0, 15-, 30-, 45-, and 60-min in a *5 mm* deep M9 buffer above an agar lawn. The linear regression equation $f = 2.38 - 0.0134t$ $(R^2 = 0.98)$ relates the average frequency (f) to time (t). (**B.**) After swimming continuously for approximately 15 minutes, a few worms altered from an undulatory to a non-propulsive coiling gait.

In summary, the likely cause of the less-than-perfect specificity is the occurrence of quiescent episodes (manuscript Fig. 3), which increase with residence time in the sorter. During these episodes, gravitaxis-competent worms are carried upward by buoyancy forces and mistakenly collected as false positives. Thus, each sorting iteration must be completed within about 20 min. To improve specificity, sorted worms can be resorted after a rest/feeding interval.

S7. Pharmacological Rescue

The MT15620 strain has a knock-out *cat-2* gene, preventing the synthesis of L-DOPA. Externally administered L-DOPA, which can be converted into dopamine in subsequent metabolic steps, can duplicate *cat-2* function. After L-DOPA treatment, MT15620 worms exhibited gravitaxis behavior.

L-DOPA, however, oxidizes upon exposure to air, and its oxidation products can be detrimental to the worms, leading to increased stress levels and injury. To minimize oxidation, we added ascorbic acid, which helps stabilize L-DOPA.

The gravitaxis-deficient behavior of the MT15620 strain was effectively restored (Table S7). As a control, we treated MT15620 worms with ascorbic acid alone, which did not rescue gravitaxis behavior, confirming that the therapeutic effect was specific to L-DOPA administration. These results are summarized in Tables S7 - S8.

S8. Fluorescent Ratio of NQ1155

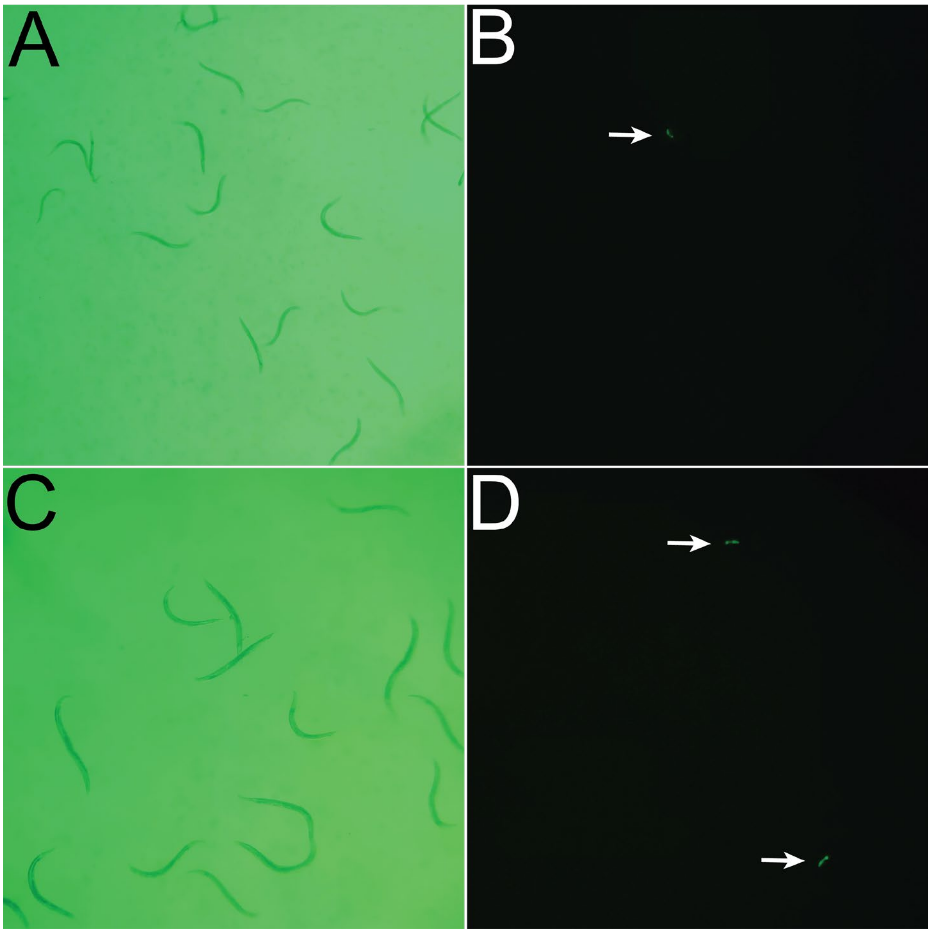

Figure S6: Stereomicroscopy images of **A**. NQ1155/MT15620 mixed sorted worms collected from the top outlet, illuminated by white light. **B**. NQ1155/MT15620 mixed sorted worms collected from the top outlet, illuminated by excitation blue light. **C**. PD4790/MT15620 mixed sorted worms collected from the top outlet, illuminated by white light. **D**. PD4790/MT15620 mixed sorted worms collected from the top outlet, illuminated by excitation blue light.

To determine sorter specificity, we performed experiments using mixed populations of gravitaxis-competent and gravitaxis-deficient worms. To distinguish between these phenotypes, we initially used the gravitaxis-competent NQ1155 strain engineered to express green fluorescence.

The GFP-encoding transgene in NQ1155 is extrachromosomal and mitotically unstable; consequently, not all progenies inherit it. To account for this, we quantified the fraction of fluorescent worms within the population. Specifically, we imaged the adult progeny of a single fluorescent NQ1155 worm under white and blue illumination to determine the percentage of progeny carrying the GFP transgene [2]. These fluorescence ratios are summarized in Table S10.

Subsequently, we obtained the GFP-expressing PD4790 strain, which was used in most of our experiments.

S9. Sorter Specificity

We define the sorter's specificity as $S_p = \frac{N_T^D}{N_T^D + N_T^C}$, where ($N_T^C$) is the number of fluorescent animals (false positives) and (N_T^D) is the number of non-fluorescent animals (true positives) discharged at the sorter's top. We introduced a blend of nearly equal numbers of gravitaxis-competent (fluorescent PD4790 or NQ1155) and gravitaxis-deficient (MT15620) worms into the sorter. After 20 minutes of sorting, we counted the number of fluorescent NQ1155 (Figs. S6A and B) or PD4790 (Fig. S6C and D) (N_T^C) and non-fluorescent NQ1115 (N_T^D). These data are summarized in Tables S11 and S12. While most gravitaxis-competent worms were collected from the bottom outlet, a small fraction was still found in the top outlet (Fig. S6).

Table S2: **N2 Sorting Efficiency**

Groups	Bottom outlet	Top outlet
1	466 (93.39%)	33 (6.61%)
2	386 (89.98%)	43 (10.02%)
3	212 (90.21%)	23 (9.79%)
4	207 (87.34%)	30 (12.66%)
5	214 (84.25%)	40 (15.75%)
6	236 (83.10%)	48 (16.90%)
Mean	88.05 ± 3.91%	11.95 ± 3.91%

Table S3: **NQ1155 Sorting Efficiency**

Groups	Bottom outlet	Top outlet
1	658 (88.80%)	83 (11.20%)
2	671 (90.80%)	68 (09.20%)
3	725 (89.95%)	81 (10.05%)
Mean	89.85 ± 1.00%	10.15 ± 1.00%

Table S4: PD4790 Sorting Efficiency

Groups	Bottom outlet	Top outlet
1	292 (81.11%)	68 (18.89%)
2	350 (88.61%)	45 (11.39%)
3	370 (83.70%)	72 (16.29%)
Mean	84.47 ± 3.81%	15.53 ± 3.81%

Table S5: MT15620 Sorting Efficiency

Groups	Bottom outlet	Top outlet
1	12 (7.45%)	149 (92.55%)
2	31 (5.85%)	499 (94.15%)
3	26 (5.62%)	437 (94.38%)
4	36 (6.88%)	487 (93.12%)
5	28 (5.49%)	482 (94.51%)
6	36 (7.11%)	470 (92.89%)
7	19 (5.05%)	357 (94.95%)
Mean	6.21 ± 0.93%	93.79 ± 0.93%

Table S6: N2 Sorting Efficiency as a Function of Sorting Time

Time (min)	Top outlet	Bottom outlet
10	41 (10.57%)	347 (89.43%)
20	12 (11.11%)	96 (88.89%)
30	24 (21.62%)	87 (78.38%)
40	38 (47.50%)	42 (52.50%)

Table S7: Sorting Efficiency of L-DOPA Rescued MT15620

Groups	Bottom outlet	Top outlet
1	235 (81.03%)	55 (18.97%)
2	245 (83.62%)	48 (16.38%)
3	194 (66.44%)	98 (33.56%)
4	179 (71.60%)	71 (28.40%)
Mean	75.67 ± 8.04%	24.33 ± 8.04%

Table S8: Sorting Efficiency of Ascorbic acid and L-DOPA Rescued MT15620

Groups	Bottom outlet	Top outlet
1	356 (90.36%)	38 (9.64%)
2	292 (89.30%)	35 (10.70%)
3	352 (87.34%)	51 (12.66%)
Mean	89 ± 1.53%	11 ± 1.53%

Table S9: Sorting Efficiency of Ascorbic acid treated MT15620 (control)

Groups	Bottom outlet	Top outlet
1	47 (9.02%)	474 (90.98%)
2	52 (17.16%)	251 (82.84%)
3	26 (10.70%)	217 (89.30%)
Mean	12.29 ± 4.30%	87.71 ± 4.30%

Table S10: NQ1155 - fluorescent ratio

Fluorescent	Total	Ratio
18	64	21.95%
17	60	22.08%
23	79	22.55%
7	52	11.86%
34	109	23.78%
30	96	23.81%
57	182	23.85%
49	170	22.37%
41	136	23.16%
53	160	24.88%
		22.03 ± 3.69%

Table S11: Sorting Specificity – a blend of NQ1155 and MT15620

**Bottom**			**Top**		
Total	NQ1155	MT15620	Total	NQ1155	MT15620
271	259 (95.57%)	12 (4.43%)	287	59 (20.56%)	228 (79.44%)
320	297 (92.81%)	23 (7.19%)	332	50 (15.06%)	282 (84.94%)
380	331 (87.11%)	49 (12.89%)	370	63 (17.03%)	307 (82.97%)
233	204 (87.55%)	29 (12.45%)	246	27 (11.00%)	219 (89.00%)
222	199 (89.64%)	23 (10.36%)	264	59 (22.35%)	205 (77.65%)
255	227 (89.02%)	28 (10.98%)	306	33 (09.73%)	273 (90.27%)
240	191 (79.58%)	49 (20.42%)	210	59 (28.10%)	151 (71.90%)
**Average**	88.75 ± 5.03%	11.25 ± 5.03%		16.20 ± 8.94%	83.80 ± 8.94%

Table S12: Sorting specificity of a mixed population of MT15620 and PD4790

**Bottom**			**Top**		
Total	PD4790	MT15620	Total	PD4790	MT15620
186	156 (83.87%)	30 (16.13%)	147	28 (19.05%)	119 (80.95%)
234	201 (85.90%)	33 (14.10%)	220	24 (10.91%)	196 (89.09%)
270	238 (88.15%)	32 (11.85%)	282	36 (12.86%)	246 (87.14%)
241	211 (84.17%)	30 (12.45%)	259	41 (16.47%)	218 (84.17%)
**Average**	86.37 ± 1.66%	13.63 ± 1.66%		14.82 ± 3.15%	85.18 ± 3.15%

Table S13: Quiescence duration in swimming N2 adults (N=304) across 10-minute intervals.

**Time interval after start of sorting (*min*)**	**Number of quiescent* episodes N_q**	**Average duration of a quiescent episode** $\Delta t_{q,k}$ (s) ± SD	**Average duration of quiescence per animal (s)****	**The ratio of average quiescent times** $\frac{\Delta t_{q,0}}{\Delta t_{q,k}} \pm S.D.$ ***
0-10	42	5.1 ± 3.9	0.7±0.5	1
10-20	55	6.1 ± 5.8	1.1±1.0	0.64±0.73
20-30	70	10.0 ± 7.5	2.3±1.7	0.30±0.31

* Quiescence is defined as the absence of motion for 1s or longer. N_q includes coiling episodes that do not produce displacement.

** $\Delta t_{a,k} = \frac{N_{q.k} \Delta t_{q,k}}{N}$

*** The standard deviation of the ratio: $\frac{\Delta t_{q,0}}{\Delta t_{q,k}} \sqrt{\left(\frac{SD_0}{\Delta t_{q,0}}\right)^2 + \left(\frac{SD_k}{\Delta t_{q,k}}\right)^2}$

Table S14: **Efficiency of repeated sorting**

Sorting Rounds	Bottom outlet	Top outlet ± SD
1	88.61 ± 4.60 %	11.39 ± 4.60 %
2	97.05 ± 1.20 %	2.95 ± 1.20 %
3	99.10 ± 0.25 %	0.90 ± 0.25 %

Supplemental References

S1. Takashi Nagashima et al., “Dopamine regulates body size in Caenorhabditis elegans”. *Developmental Biology* 412.1 (2016), pp. 128–138.

S2. Craig C Mello et al., “Efficient gene transfer in C. elegans: extrachromosomal maintenance and integration of transforming sequences”. *The EMBO journal* 10.12 (1991), p. 3959.

S3. Rafael D Schulman et al. “Dynamic force patterns of an undulatory microswimmer,” *Physical Review E* 89.5 (2014), p. 050701.

S4. Wan-Jung Kuo, Yue-Syun Sie, and Han-Sheng Chuang, “Characterizations of kinetic power and propulsion of the nematode Caenorhabditis elegans based on a micro-particle image velocimetry system”. *Biomicrofluidics* 8.2 (2014).

S5. James Gray and Gregory J Hancock, “The propulsion of sea-urchin spermatozoa”. *Journal of Experimental Biology* 32.4 (1955), pp. 802–814.
